# Diagnostic Decision-Making Variability Between Novice and Expert Optometrists for Glaucoma: Comparative Analysis to Inform AI System Design

**DOI:** 10.2196/63109

**Published:** 2025-01-29

**Authors:** Faisal Ghaffar, Nadine M. Furtado, Imad Ali, Catherine Burns

**Affiliations:** 1 Department of Systems Design Engineering Faculty of Engineering University of Waterloo Waterloo, ON Canada; 2 School of Optometry and Vision Science University of Waterloo Waterloo, ON Canada; 3 Department of Computer Science University of Swat Mingora Pakistan

**Keywords:** decision-making, human-centered AI design, human factors, experts versus novices differences, optometry, glaucoma diagnosis, experts versus novices, glaucoma, eye disease, vision, vision impairment, comparative analysis, methodology, optometrist, artificial intelligence, AI, diagnostic accuracy, consistency, clinical data, risk assessment, progression analysis

## Abstract

**Background:**

While expert optometrists tend to rely on a deep understanding of the disease and intuitive pattern recognition, those with less experience may depend more on extensive data, comparisons, and external guidance. Understanding these variations is important for developing artificial intelligence (AI) systems that can effectively support optometrists with varying degrees of experience and minimize decision inconsistencies.

**Objective:**

The main objective of this study is to identify and analyze the variations in diagnostic decision-making approaches between novice and expert optometrists. By understanding these variations, we aim to provide guidelines for the development of AI systems that can support optometrists with varying levels of expertise. These guidelines will assist in developing AI systems for glaucoma diagnosis, ultimately enhancing the diagnostic accuracy of optometrists and minimizing inconsistencies in their decisions.

**Methods:**

We conducted in-depth interviews with 14 optometrists using within-subject design, including both novices and experts, focusing on their approaches to glaucoma diagnosis. The responses were coded and analyzed using a mixed method approach incorporating both qualitative and quantitative analysis. Statistical tests such as Mann-Whitney *U* and chi-square tests were used to find significance in intergroup variations. These findings were further supported by themes extracted through qualitative analysis, which helped to identify decision-making patterns and understand variations in their approaches.

**Results:**

Both groups showed lower concordance rates with clinical diagnosis, with experts showing almost double (7/35, 20%) concordance rates with limited data in comparison to novices (7/69, 10%), highlighting the impact of experience and data availability on clinical judgment; this rate increased to nearly 40% for both groups (experts: 5/12, 42% and novices: 8/21, 42%) when they had access to complete historical data of the patient. We also found statistically significant intergroup differences between the first visits and subsequent visits with a *P* value of less than .05 on the Mann-Whitney *U* test in many assessments. Furthermore, approaches to the exam assessment and decision differed significantly: experts emphasized comprehensive risk assessments and progression analysis, demonstrating cognitive efficiency and intuitive decision-making, while novices relied more on structured, analytical methods and external references. Additionally, significant variations in patient follow-up times were observed, with a *P* value of <.001 on the chi-square test, showing a stronger influence of experience on follow-up time decisions.

**Conclusions:**

The study highlights significant variations in the decision-making process of novice and expert optometrists in glaucoma diagnosis, with experience playing a key role in accuracy, approach, and management. These findings demonstrate the critical need for AI systems tailored to varying levels of expertise. They also provide insights for the future design of AI systems aimed at enhancing the diagnostic accuracy of optometrists and consistency across different expertise levels, ultimately improving patient outcomes in optometric practice.

## Introduction

Artificial intelligence (AI) has emerged as a transformative force in optometry [1], particularly for glaucoma diagnosis. In this context, AI in optometry refers to the advanced algorithms that analyze complex data such as fundus photos and related patient data. These AI systems use techniques of image processing, computer vision, deep learning, and transfer learning to detect and diagnose glaucoma. Glaucoma is a progressive eye disease that often eludes early detection due to its subtle or absent symptoms, which poses a unique challenge for optometrists [2]. This challenge, along with no clear-cut tests or signs, frequently results in variability in glaucoma-related decisions, influenced by factors such as the optometrists’ expertise, experience, the subjective interpretation of clinical data, and the use of different equipment [3,4]. This variability is a critical issue that AI is well positioned to address, potentially standardizing the diagnostic process and offering a more objective analysis of clinical data. Studies have illustrated AI’s advanced analytical capability and proficiency in sifting through extensive patient data, including retinal images, visual field tests, optical coherence tomography scans, and medical histories [5]. This advanced analytical capability enables AI to identify signs of glaucoma with consistency and accuracy, often surpassing that of human optometrists [6,7]. For example, in Akter et al [8], researchers found that AI algorithms could detect glaucoma with up to 96% accuracy, markedly higher than the 80% accuracy rate commonly associated with optometrists.

Similarly, in another study [9], an accuracy of 83.4% is reported in identifying glaucomatous optic neuropathy using AI. These findings show AI’s potential to reduce misdiagnoses and address the prevalent decision variability. In a similar vein, in another study [10], it was demonstrated that an AI system helped junior radiologists perform at levels close to that of a senior radiologist. This finding is particularly relevant to optometry, with AI having the potential to play a similar role in enhancing the diagnostic abilities of less experienced optometrists. As in Jammal et al [11], researchers have highlighted that advocating for a more integrated use of AI tools in optometric practices could be a significant step toward standardizing care and reducing diagnostic variability.

However, the effective integration of AI into optometric practice hinges on a comprehensive understanding of optometrists’ decision-making processes [12,13]. AI promises to offer standardization in clinical decision-making. Yet, the crux of its successful integration lies in deeply comprehending the subtle intricacies inherent in human decision-making. This understanding is especially critical in diagnosing conditions like glaucoma, where subjective assessments and clinical expertise play significant roles. Highlighting the variability in diagnostic approaches between novice and expert optometrists, as demonstrated by studies [14,15], shows the importance of tailored AI support. Therefore, AI systems must adapt to different levels of clinical expertise and integrate the diverse decision-making processes of novice and expert optometrists.

Diagnostic decisions in this field vary significantly, influenced by an optometrist’s expertise, experience, and cognitive strategies. These differences affect how patient data are interpreted, the importance assigned to various clinical findings, and the resulting diagnostic outcomes.

In this study, we investigate the variance of diagnostic decisions made by optometrists in the context of glaucoma, focusing on how the availability and nature of data influence these decisions. Several key research questions guide our investigation.

1. How do the decision-making processes of expert and novice optometrists differ when they are presented with the same patient data?
2. What specific data do optometrists at different experience levels tend to prioritize in a clinical journey?
3. Are experienced optometrists more inclined to use heuristic approaches when confronted with limited data instead of scenarios with comprehensive data availability?

To address our research questions, we conducted in-depth interviews with novice and expert optometrists, focusing on their diagnostic approaches to glaucoma. Following these interviews, we performed a detailed coding of the responses, enabling us to conduct qualitative and quantitative analyses. This structured approach allowed us to comprehensively examine the decision-making patterns and their implications for AI integration in optometric practice.

Our research aims to comprehensively understand how optometrists make decisions when diagnosing glaucoma, exploring the diversity in their approaches. By exploring the variations in decision-making between novice and expert optometrists, our study aims to pave the way for the future design of AI systems. These envisioned systems are intended to bridge the expertise gap in glaucoma diagnosis. They will complement human judgment to reduce biases and enhance diagnostic accuracy, leading to more consistent and reliable evaluations. The insights gained from this exploration are anticipated to significantly contribute to optometric practice and education, setting the stage for future advancements in glaucoma diagnosis and management.

## Methods

### Overview

**Figure 1:**
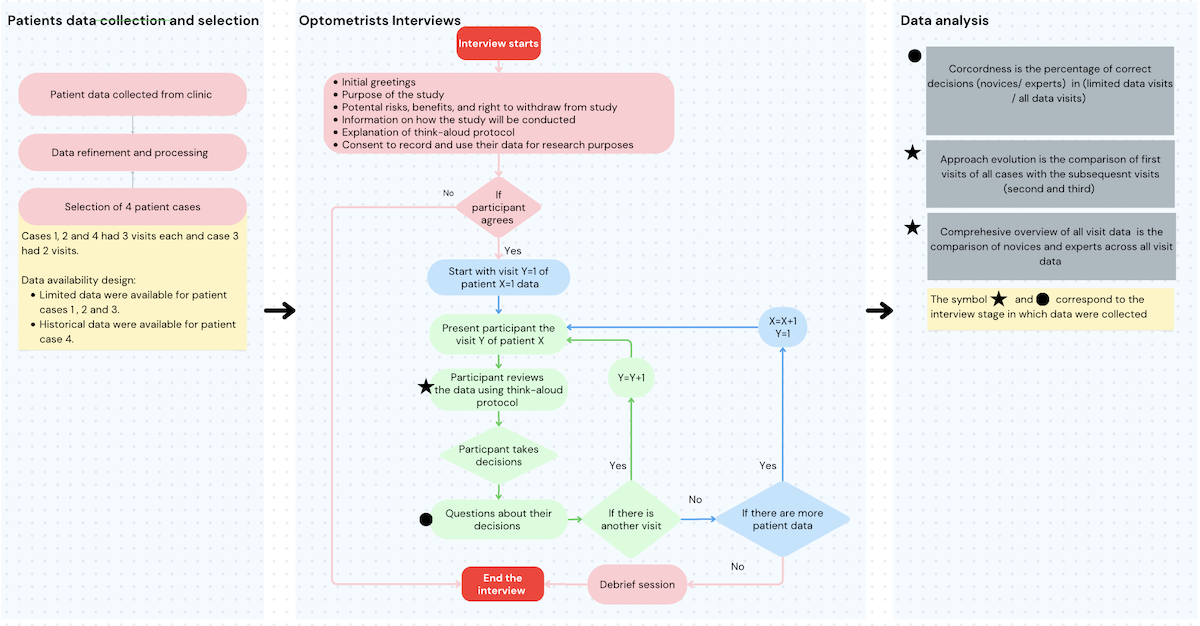
The stages and flow of the study. It started with patient data collection and selection, followed by optometrist interviews and data analysis. Each stage is further subdivided into steps. Each of these steps is further explained in the Methods section. The circle and star symbols show the data obtained at a specific stage in the interview and how they were analyzed. A higher resolution version of this image is included as Multimedia Appendix 1.

This section details our research methodology investigating variations in optometrists’ decision-making. It begins with an in-depth explanation of the data collection process, emphasizing the criteria and methods used for selection. Subsequently, the stages of data refinement and processing are outlined. Optometrist interviews are discussed, covering outreach strategies, interview platforms, and participant demographics. A within-subjects design was used for the interviews, allowing each participant to engage with all patient cases. The section culminates with insights into the data coding process, involving transcript cleaning and multidisciplinary analysis. For the data analysis, a mixed methods approach was used, integrating both qualitative and quantitative data to provide a comprehensive understanding of the decision-making processes. Figure 1 summarizes the flow of the study.

### Ethical Considerations

This study (application 43288) was reviewed and approved by the Delegated Research Ethics Committee (DERC) of the University of Waterloo on May 27, 2021. Informed consent was sought from the participants before the data collection. They were informed about the procedure, purpose, benefits, risks, and their right to withdraw from the study. Further, they were informed that data acquired from the interview would be anonymized, deidentified, stored on a protected server, and used anonymously in all publications that arise from the study. Participants were remunerated CAD $150 (CAD $1=US $0.75) for their 2-hour commitment, recognizing their professional contribution and encouraging detailed participation.

### Patients’ Data Collection

Data for this study were acquired from the School of Optometry and Vision Sciences at the University of Waterloo, Canada. The selection process entailed scrutiny of patient records within the institution’s database, explicitly targeting those records wherein patients had granted explicit consent for their information to be used in research. The preliminary phase of our data collection involved gathering records from 8 distinct patients. Each patient’s record provided a longitudinal perspective, encompassing multiple clinical visits and offering an in-depth view of their interactions with the optometrist over time.

The data attributes collected from patient records were informed by research from previous similar studies [16-19] and the European Glaucoma Prevention Study [20]. Our data collection encompasses a comprehensive range of patient characteristics. It includes essential ocular health metrics and relevant external factors to ensure a comprehensive glaucoma evaluation by optometrists. The patient features we collected are detailed in Table 1. These features reflect the multifaceted factors highlighted in the referenced studies.

**Table 1:** Data features for glaucoma diagnosis collected from the electronic health record system from the School of Optometry, University of Waterloo.

Category	Factors
Demographic factors	Age, gender, and race
Historical data	Ocular history, medical history, medication history, social history, and family history
Internal attributes	Habitual spectacle prescription, unaided visual acuity, aided acuity, pupils, anterior segment examination, Goldman applanation tonometry, central corneal thickness, and gonioscopy
Images	Fundus image and OCT^a^ images
Reports	Posterior segment examination, visual field, and OCT

^a^OCT: optical coherence tomography.

### Data Refinement and Processing

The data refinement and processing were executed to facilitate the interview component of the study. Out of the initial pool of patients, 4 records were chosen to be reviewed for the in-depth interviews. This selection process involved comprehensive discussions with an expert in the field to identify cases that could provide diverse diagnostic perspectives. The criteria for case selection were centered on identifying instances that would likely elicit differing opinions among optometrists, thereby enabling a focused examination of the variation in clinical decision-making.

The rationale behind limiting the selection to 4 patient records stemmed from practical constraints associated with the review process. It was anticipated that participants would take about 10-15 minutes to review each patient visit. To manage this effectively within time constraints, we selected 3 patients with 3 visits (the initial, final, and intermediary visits) and 1 patient with 2 visits. This approach allowed for a detailed review of each patient’s clinical journey while staying within the practical limits of the allotted time. Notably, historical data from previous visits were not provided for 3 of the 4 patients. However, more exhaustive data were provided for 1 patient, including the complete set of previous visits data, offering a more detailed case study within the research. This approach provided a balanced perspective between a broad overview and an in-depth case analysis, enhancing the study’s ability to capture general trends and specific nuances in clinical decision-making.

### Optometrist Interviews

In this study, we interviewed 14 optometrists from the Waterloo region, primarily affiliated with the University of Waterloo’s School of Optometry and Vision Science. Each optometrist participated in a 100- to 120-minute, web-based interview conducted in English via Microsoft Teams and Zoom. The choice of web-based interviews over physical ones was in response to the participants’ preferences.

The participants had an average of 7.54 (SD 8.34) years of experience and saw an average of 32.57 (SD 22.39) patients weekly.

Each optometrist scrutinized data from at least 10 patient visits, translating to a comprehensive review spanning about 2 hours for the entire set of patient records. Although we aimed to present all visit data for evaluation, time constraints necessitated the omission of some visits, and a few evaluations did not produce usable data. Throughout the interviews, participants employed the “think aloud” protocol to review patient records, aiding in understanding their decision-making processes. After the first 10 interviews, we observed data saturation, which was achieved by the 14th interview, as evidenced by recurring themes and no new substantial information. All interviews were audio-recorded and transcribed using the platforms’ built-in features.

### Data Coding

Our qualitative research commenced with the cleaning of interview transcripts. This preliminary step involved a thorough review in correcting errors, eliminating irrelevant sections, and enhancing clarity and coherence. This crucial stage ensured the data’s integrity before proceeding to the analysis phase.

After cleaning, the refined transcripts were exported into NVivo (version 14.23.2; Lumivero, formerly QSR International). In NVivo, we carried out an open coding process led primarily by FG. The involvement of multidisciplinary expertise enriched the coding process. With a background in engineering, CB conducted a thorough review of the codes from an engineering perspective. This review added a layer of technical and analytical insight, ensuring that the coding accurately reflected relevant engineering concepts and terminologies. Simultaneously, NF, leveraging her expertise in optometry, reviewed the codes with a focus on the optometry perspective. Her review was pivotal in aligning the data with optometric principles and practices, ensuring the findings were relevant and accurately interpreted within the context of eye care and vision science.

This multidisciplinary approach to reviewing the codes enhanced the depth of our analysis and ensured that the findings were robust, well rounded, and reflective of diverse professional viewpoints. Such a collaborative effort was instrumental in ensuring that the themes and patterns identified were relevant and applicable across different fields of study.

The open coding was executed inductively, allowing the data to drive the categorization process rather than imposing preexisting theories or frameworks. This approach facilitated a more organic and grounded understanding of the data. Each transcript line was carefully examined, with open codes assigned to summarize the content succinctly. These codes were crafted to stay true to the original text, minimizing abstraction. This level of descriptiveness in coding was instrumental in capturing the essence of the participants’ narratives, laying a solid foundation for subsequent conceptualization and analysis. The codes were then grouped to form more generalized codes. Multimedia Appendix 2 gives details of the created generalized codes.

Through this process, we distilled the raw data into meaningful themes and patterns, shedding light on the underlying narratives and insights embedded within the transcripts.

### Data Analysis

To carry out the analysis, we categorized participants into 2 groups: novices and experts, based on their years of experience and frequency of consultations with patients with glaucoma, using median values for greater precision. An “expert” was defined as having 4 or more years of experience as a practicing optometrist and treating a higher-than-average volume of patients with glaucoma per week compared with the participant pool. This classification excluded the average number of all patients examined or treated in a week for any eye condition, as it was deemed irrelevant in the context of glaucoma. Participants collectively conducted 137 evaluations, with experts reviewing 48 and novices reviewing 89. Ideally, the total number of evaluations would have been 144, but in some visit instances, the participants did not provide us with enough data, or we skipped some visit instances in the interview because of time constraints.

First, we examined the variability of novices and experts in diagnostic concordance across all patient visits, focusing on how the availability of comprehensive versus missing data influences concordance rates. Diagnostic concordance is the rate of optometrists’ decisions that are aligned with the original clinical decision taken at a clinic. This analysis revealed disparities between optometrists’ decisions and established clinical decisions. Following this analysis, we examined the approach evolution of optometrists within the clinical journey. This analysis was conducted by calculating the assessments/exams conductance rate (the percentage of a specific assessment conducted) and 95% CI, and performing statistical analysis using Mann-Whitney *U* [21] test to evaluate the significance of the variations and approach evolution from initial visits to subsequent visits. To observe a general trend of evolution, we first compared and analyzed initial visits (n=51) with follow-up visits (n=82). To observe the novice and experts’ trends, we then further classified the data by experience level of optometrists, comparing initial (n=33) and follow-up (n=55) visits by novices to initial (n=18) and follow-up (n=27) visits by experts. Through this analysis, we uncovered the variation in approach evolution and clinical decision-making between novices and expert optometrists.

Second, we used quantitative and qualitative methodologies across all evaluations (N=137) to gain an in-depth understanding of the outcomes of these examinations. This approach enabled us to identify key themes that distinguish the ocular examination practices of novice and expert optometrists. Similar to earlier analysis, we calculated the assessments/exams conductance rate and 95% CI, and performing statistical analysis using Mann-Whitney *U* test to compare each assessment type of experts and novices. These findings were further supported by identified themes. This mixed method analysis provided insights into the consistency and variability in assessments and diagnostic reasoning. Moreover, by intercase and intracase comparison analysis, we identified the cognitive strategies in clinical decision-making, revealing how expertise level influences the patient case comparison.

Last, we calculated the average follow-up time of experts and novices and then used chi-square test to observe the significance of variation. This analysis offered an understanding of how experience impacts the timelines of patient management. This finding is crucial in glaucoma, where timely intervention can significantly influence disease progression and patient quality of life.

Overall, our study provides a multifaceted look at the complexities of glaucoma management, highlighting the influence of experience on various aspects of patient care. These insights are invaluable for shaping future educational programs, clinical guidelines, and AI systems in glaucoma, ensuring that they are tailored to address the specific needs and challenges optometrists face.

## Results

### Variability in Diagnostic Concordance and Approach Evolution in the Clinical Journey

This section investigates the variability in diagnostic concordance among novice and expert optometrists relative to established clinical decisions, emphasizing the influence of accessible patient data on diagnostic accuracy. It also delineates the evolution of diagnostic and treatment methodologies throughout the clinical journey, from initial patient encounters to subsequent visits.

Variability exists in the diagnostic concordance and approach evolution between experts and novices throughout the clinical journey. We compared the diagnostic decisions made by our participants with established clinical decisions, which we define as the decisions previously made by optometrists in a clinical setting where they had access to additional patient data, including comprehensive medical histories and previous examination results. The diagnostic concordance measures the extent to which our participants’ decisions align with these preexisting clinical decisions. We observed significant variations in the decision-making of optometrists, notably when they lacked prior patient data.

Key findings highlight the disparity in diagnostic decisions when previous patient history is not available, where compared with novices (10% concordance, 7/69 evaluations), experts showed a higher rate of concordance (20% concordance, 7/35 evaluations), highlighting the impact of experience on clinical judgment. Interestingly, when provided with prior patient data, the gap in decision-making accuracy between experts (42% concordance, 5/12 evaluations) and novices (42% concordance, 8/21 evaluations) decreased. This finding shows the critical role of comprehensive patient histories in enhancing diagnostic precision, particularly for less experienced optometrists.

**Figure 2:**
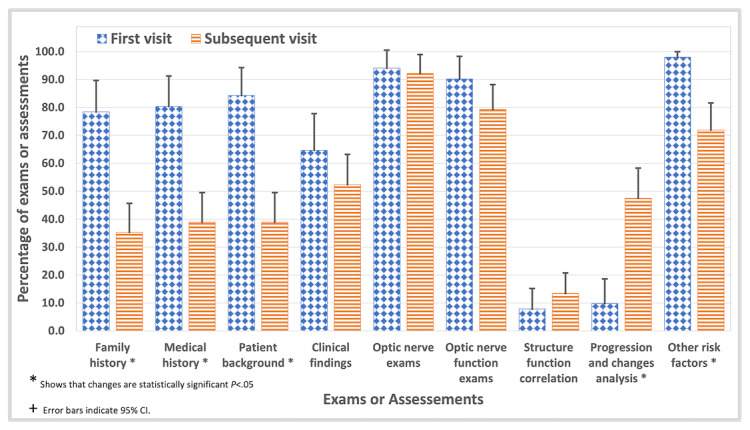
The percentage increase or decrease in glaucoma-related eye examinations and assessments conducted by optometrists, tracked from the initial visit to subsequent visits through longitudinal patient history.

Building upon the insights regarding diagnostic concordance, the optometrists’ diagnostic approaches through the clinical journey of a patient reveal significant differences and shifts in practice strategy and emphasis. Initially, there is a higher engagement across all assessments except progression and change analysis and structure and function correlation analysis reflecting common practice for initial assessments. However, in subsequent visits, data reveal a significant shift: the engagement with most assessments decreased, while progression and change, and structure and function correlation, increased. Figure 2 provides a graphical representation of these trends in percentage, illustrating the shift from broad-based examinations to focused analytical assessments. Further analysis using the Mann-Whitney *U* test showed that these changes were significant when comparing initial and subsequent visits. Significant differences were observed in family history (*P*<.001, Cohen *d*=0.41), medical history (*P*<.001, Cohen *d*= 0.45), patient background (*P*<.001, Cohen *d*=0.43), other risk factors (*P*<.001, Cohen *d*=0.50), clinical findings (*P*=.01, Cohen *d*=0.28), optic nerve exams (*P*=.01, Cohen *d*=0.29), as well as in progression and change analysis (*P*=.005, Cohen *d*=–0.20). There was no significant change observed in the optic nerve function exam and structure-function correlation assessments. The shift in engagement with different assessments shows a pivot towards more in-depth analytical work.

To investigate further, we compared and analyzed the data of experts and novices separately, summarized in Table 2. Detailed descriptive statistics are provided in Multimedia Appendix 3. In the initial visit, novices emphasize direct examination techniques, focusing significantly on the optic nerve exam (31/33, 94%) and optic nerve function exam (30/33, 91%) and comparatively underemphasizing patient history and background information, while experts demonstrate a more balanced and comprehensive approach. They engage more deeply in gathering medical history (15/18, 83%) and patient background (17/18, 94%), while still maintaining high attention to optic nerve assessments (16/18, 88.8%). Both give considerable attention to other risk factors, with experts at 100% (18/18) and novices at 97% (32/33). These results suggest that experts prioritize a more complete picture of the patient’s medical background and current health status from the onset. Regarding the analytical methods such as progression and change analysis, novices show a surprising engagement (4/33, 12%), which demonstrates a higher propensity to engage in advanced analytical methods early on, despite such analyses typically requiring follow-up data. This is possibly due to a misunderstanding of the optimal timing for such analyses.

**Table 2:** The variation in glaucoma-related eye examinations throughout a patient’s clinical journey, highlighting the differences between novice and expert optometrists, tracked from initial to subsequent visits. For the Mann-Whitney U test, effect size is given by the rank biserial correlation.

	Novices				Experts			
	First visit, n/N (%)	Subsequent visit, n/N (%)	*P* value	Cohen *d*	First visit, n/N (%)	Subsequent visit, n/N (%)	*P* value	Cohen *d*
Family history	26/33 (79)	19/55 (35)	<.001	.39	14/18 (78)	10/27 (37)	.02	0.41
Medical history	26/33 (79)	21/55 (38)	<.001	.44	15/18 (83)	11/27 (41)	.01	0.47
Patient background	26/33 (79)	18/55 (33)	<.001	.53	17/18 (94)	14/27 (52)	.03	0.29
Clinical findings	19/33 (58)	23/55 (42)	.04	.27	14/18 (78)	20/27 (74)	.63^a^	0.09
Optic nerve exams	31/33 (94)	48/55 (87)	.01	.36	16/18 (89)	25/27 (93)	.35^a^	0.18
Optic nerve function exams	30/33 (91)	44/55 (80)	.74^a^	.04	16/18 (89)	21/27 (78)	.80^a^	–0.04
Structure-function correlation	2/33 (6)	4/55 (7)	.51^a^	–0.05	2/18 (11)	7/27 (26)	.21^a^	–0.17
Progression and change analysis	4/33 (12)	15/55 (27)	.68^a^	–0.04	1/18 (6)	24/27 (89)	<.001	–0.7
Other risk factors	32/33 (97)	41/55 (75)	<.001	0.5	18/18 (100)	18/27 (67)	.06^a^	0.35

^a^Nonsignificant values.

In the subsequent visits shown in Table 2, there is a noticeable shift. Novices demonstrated sustained attention to optic nerve exams (48/55, 87%) and optic nerve function exams (44/55, 80%). However, their assessment of family history, medical history, patient background, and other risk factors dropped in comparison to that of initial visits. Experts showed a similar behavior with high attention to optic nerve exams (25/27, 92%), optic nerve function exams (21/27, 77%), and clinical findings (20/27, 74%), while showing a marked decrease in the assessment of family history, medical history, patient background in comparison to initial visits. In the assessment of progression and changes analysis, and structure-function correlation, both groups showed an increase. However, the extent of these increases differs between the 2 groups; novices showed a modest increase in progression and changes assessment (15/55, 27%) and a much smaller increase in structure function correlation (4/55, 7%), while experts showed a robust increase (24/27, 88%) in progression and changes assessment and modest increase (7/27, 25%) in structure-function correlation. This leap for experts could reflect a strategic approach where they dive deeper into the more complex analysis. This finding might suggest a more focused approach as specific patient issues become more apparent.

Further analysis using the Mann-Whitney *U* test showed significant shifts in the assessments from initial to subsequent visits for both novices and experts, as reported in Table 2. In this analysis, the weight of each exam was also considered and calculated based on the frequency with which an optometrist discussed a specific assessment. We observed that novices showed significant changes in more assessments with Cohen *d* values of moderate effect, suggesting a strong shift of approach. While the experts showed fewer significant changes with Cohen *d* values of smaller to moderate effect, except for the progression and change analysis where the effect was large, suggesting a stronger shift. These findings suggest that experts maintained a comprehensive approach, balancing all assessments with increased emphasis on analytical assessment.

This analysis highlights the critical importance of comprehensive patient data in enhancing diagnostic accuracy, particularly for novice optometrists who face more significant challenges without extensive patient histories. The findings reveal the dynamic evolution of diagnostic and treatment strategies throughout a patient’s clinical journey, illustrating how adaptability in practice strategy from initial assessments to subsequent visits is crucial. Notably, novices demonstrate a reduced focus on disease progression and correlating variables, indicating a gap in their approach to long-term patient management. This result highlights the potential need for a guiding system to help novices navigate the complexities of diagnostic decisions more effectively.

### Comprehensive Overview of Examination Data Across All Visits

We categorized the analyses carried out by optometrists into 9 broad codes/categories: family history, medical history, patient background, clinical findings, optic nerve exams, optic nerve function exams, structure-function correlation, progression and change analysis, and other risk factors. The details of these categories are provided in Multimedia Appendix 2. Through these categories, we identified the following key themes that gave us insights into the factors that optometrists keep in focus while screening patients for glaucoma.

### All Optometrists Focus More on Optic Nerve Exams and Risk Factors

**Figure 3:**
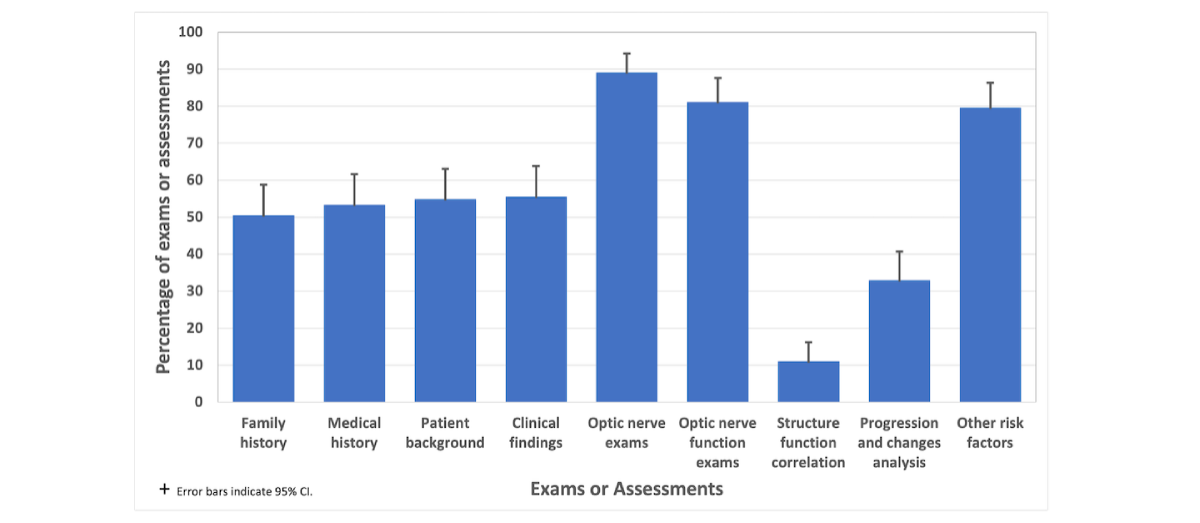
Findings from the whole dataset, revealing a strong emphasis on optic nerve structural and functional assessments. It also shows moderate attention to clinical findings, medical history, and family history, while progression analysis and structure-function correlation analysis were performed less frequently.

The data reveal the distribution of eye examinations, as shown in Figure 3, within the whole data set. Key findings include a high frequency of focus on the optic nerve assessment (122/137, 89%) and optic nerve function assessment (110/137, 80.2%), showing a significant focus on assessing the optic nerve’s structural and functional aspects. Clinical findings (75/137, 54.7%), medical history (73/137, 53.2%), and family history (69/137, 50.3%) were evaluated at moderate rates, indicating a holistic approach that incorporates patient-related and clinical information. In contrast, progression and changes analysis (45/137, 32.8%) and structure-function correlation analysis (14/137, 10.2%) were less frequently performed.

### Experts Carry Out Progression and Change Analysis More Than Novices

**Figure 4:**
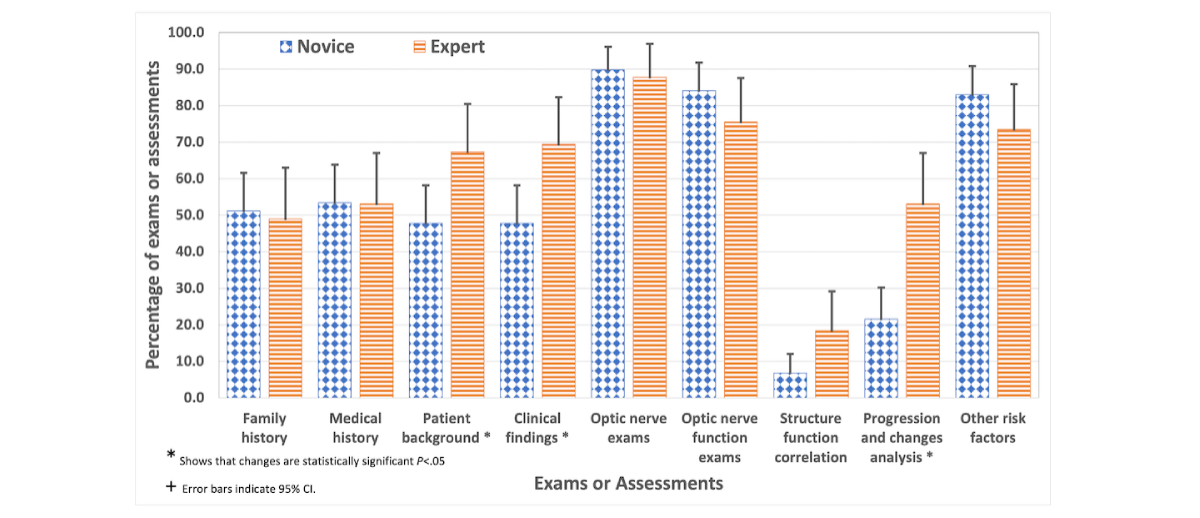
The percentages of glaucoma-related eye examinations conducted by novices and experts, highlighting differences in focus on clinical findings, patient history, progression and change analysis, and structure-function correlation.

Our findings reveal that experts are more inclined to engage in progression and change analysis than novices. A preliminary test of normality on the data distribution resulted in a *P*<.001, indicating a deviation from normal distribution. Consequently, we used the Mann-Whitney *U* test to examine the differences between experts and novices in analyzing progression and changes. This test yielded a *P*<.001 (2-tailed) with *U* of 1486, and a mean rank of 61.08 for novices and 80.54 for experts. This shows a statistically significant difference between the 2 cohorts. Specifically, our analysis shows that experts demonstrate a markedly higher focus on progression and change analysis (26/49, 53.1% vs 19/88, 21.5%), as illustrated in Figure 4, reflecting their seasoned approach to monitoring disease development over time.

### Experts Integrate Clinical Findings and Patient Background More Than Novices

There is a significant difference in the emphasis on clinical findings and patient background between novice and expert optometrists. Our initial analysis involved conducting tests of normality for both clinical findings and patient background data, which revealed *P*<.001, indicating that the data do not follow a normal distribution. Consequently, we applied the Mann-Whitney *U* test to assess the differences in emphasis on these diagnostic components between novice and expert optometrists. The test results for clinical findings yielded a *P* value of .04 (2-tailed) with *U* of 1695 and a mean rank of 63.75 for novices and 77.19 for experts. Same way the test results for patient background yielded a *P* value of .04 (2-tailed) with *U* of 1732 and a mean rank of 64.18 for novices and 76.42 for experts, suggesting a statistically significant difference between the 2 groups, albeit with a modest significance level.

Specifically, novices consider both factors in 48% (42/88) of evaluations. In contrast, experts incorporate them at a higher rate, with about 69% (34/49) for clinical findings and 67% (33/49) for patient background, as shown in Figure 4. This disparity shows the experts’ more substantial focus on combining clinical signs and symptoms of glaucoma with the patient’s historical data. Expert physicians’ comprehensive approach showcases their in-depth understanding and extensive experience in evaluating these essential diagnostic components.

### Experts Tend to Emphasize Structural Function Correlation More Than Novices

Our analysis shows a trend that novices focus on structure-function correlation at a rate of 7% (6/88) and experts at a higher rate of 18% (9/49), as depicted in Figure 4. Despite the apparent disparity in percentages suggesting a more pronounced awareness of the interplay between structural and functional considerations, statistical analysis reveals that the difference in structure-function correlation between experts and novices does not achieve statistical significance (*P*=.15 with *U*=1951.5). However, the percentage difference still indicates that there may be a trend towards a higher engagement with structure-function correlation among experts than novices.

### Novices Exhibit a Higher Tendency for Intercase and Longitudinal Comparisons, Less for Intracase Comparisons

In such a comparison, the clinician evaluates the current case against other patient scenarios. Novices in some cases made a reference to another patient’s data, denoting a reliance on external case references. This reliance may be a compensatory mechanism for the relative inexperience in glaucoma diagnosis, where pattern recognition is still being cultivated. In a representative case, a novice commented “I think that they would be potentially a mild suspect compared to the first patient that we had just due to the fact that this person has hypertension, and they have a family history of glaucoma...”

In stark contrast, this form of comparison was entirely absent among experts, suggesting a transition towards an intuitive, more synthesized comprehension of glaucoma presentations, informed by an extensive repository of patient encounters.

The difference between experts and novices in conducting longitudinal comparisons of patients’ data is subtle, with our analysis revealing a difference that does not reach statistical significance with *P*>.05. Despite the lack of statistical significance, the observed percentages 24% (12/49) of evaluations for experts versus 29% (26/88) for novices hint at an interesting trend: experts might possess a nuanced internalization of a patient’s historical data. This suggests that experts are potentially more adept at retaining key details of disease progression within their working memory. With their extensive experience, they may draw upon a mental synthesis of the patient’s history and current presentation without the need for frequent referrals to past records. This cognitive efficiency reflects a mastery of pattern recognition characteristic of glaucomatous progression, allowing for a more agile and comprehensive assessment that leverages their extensive experience and nuanced understanding of the disease. The less frequent engagement with longitudinal comparison by expert optometrists may not indicate a disregard for the patient’s history but rather an advanced capacity to retain and integrate this information into their clinical judgments, a testament to their evolved diagnostic expertise.

Intracase comparisons, particularly the evaluation of asymmetry between left and right eye examinations, were used by novices in 17% (15/88) of evaluations and experts in 24% (12/49) of evaluations with no statistically significant difference. Both groups recognize the diagnostic value of these comparisons, yet the slightly higher use by experts may reflect a refined, systematic approach and a heightened sensitivity to the subtleties of asymmetry, honed through repeated clinical encounters.

### Variation in Patient Follow-Up Time Suggestion

The analysis reveals significant variations in follow-up times among all participating optometrists, as well as a noticeable relationship with their years of experience. The initial chi-square statistic (*χ*^2^_98_=142.2) with a *P* value of .002 highlights a substantial difference in follow-up times among all optometrists. Further analysis, considering the years of experience, shows a chi-square statistic (*χ*^2^_140_=1540) with a *P* value of <.001, suggesting that experience level is an influential factor in this variation. These data collectively indicate that not only do follow-up times vary among optometrists, but they also correlate significantly with their professional experience.

A further breakdown shows that expert optometrists, with an average follow-up time of 4.1 months for confirmed glaucoma cases and 4 months for suspected glaucoma cases, seem to require less time to assess disease progression, likely due to their proficiency in managing the condition. In contrast, novice optometrists recommend longer follow-up times, averaging 5.3 months for confirmed cases and 4.5 months for suspected cases, which may indicate a need for additional time to make informed decisions about disease progression. For patients without glaucoma, both groups suggest more extended follow-up periods, with experts recommending an average of 8.15 months and novices slightly more at 8.73 months.

## Discussion

### Overview

This study investigates how optometrists’ experience levels affect their decision-making in glaucoma diagnosis, a crucial factor for patient outcomes. We found significant differences in diagnostic approaches between novices and experts. The discussion ahead will dive deeper into these differences in decision-making, particularly focusing on how experience levels affect priorities in data interpretation and heuristic use in diagnosis. This analysis will not only correlate our findings with the central research questions but also inform the development of AI systems tailored for glaucoma diagnosis.

### Decision-Making Processes

Our findings reveal a notable divergence in the alignment with established clinical decisions between expert and novice optometrists, particularly in the absence of prior patient data, offering crucial insights into the role of experience in clinical judgment. The higher alignment of expert diagnoses with original clinical decisions in scenarios lacking previous patient data highlights the depth of expertise honed through years of clinical practice. This expertise results in more accurate and intuitive diagnostic skills. This aligns with Hammond Cognitive Continuum Theory [22], which posits that as clinicians gain experience, their decision-making process shifts from a predominantly analytical approach to a more intuitive one [23,24]. Experienced practitioners are theorized to navigate and use this spectrum effectively, adapting their approach depending on the situation [25].

The reduced gap in decision-making accuracy when prior patient data were provided suggests that access to comprehensive patient history significantly enhances diagnostic accuracy across experience levels highlighting the critical role of patient history in clinical decision-making. These results suggest that novices can make competent decisions when guided by detailed information.

### Data Prioritization in Clinical Assessments

The study also explored how optometrists at different experience levels prioritize data during clinical assessments. Initially, both experts and novices engaged comprehensively across various diagnostic areas, but with a lower focus on progression, change, and correlation analysis. This trend reflects common practice for initial assessments, where establishing a broad understanding of the patient’s condition is prioritized over detailed analysis.

Interestingly, subsequent visits revealed a significant increase in attention to progression and change, particularly among experts. Experts demonstrated a more comprehensive approach, integrating clinical findings and patient history from the outset and emphasizing structural-function correlation more than novices. This comprehensive risk assessment aligns with the notion that experienced clinicians can synthesize complex information more effectively [23] and that is why their concordance was quite higher than novices.

Novices, despite a higher initial engagement progression and change analysis, might misunderstand the optimal timing for such analyses, typically requiring follow-up data. Although this increased for novices in subsequent visits, the experts showed a robust increase almost double that of novices. This indicates a strategic approach to deeper analysis in the later stages of patient management by experts.

### Heuristic Approaches and Cognitive Efficiency

Our results suggest that experienced optometrists may use heuristic approaches more frequently, particularly when data are limited. The nuanced internalization of a patient’s historical data by experts, evidenced by their subtle differences in conducting longitudinal comparisons and intercase comparisons, suggests cognitive efficiency. This efficiency allows experts to retain and integrate key details of disease progression into their clinical judgments without frequent referrals to past records or references to other cases. This cognitive agility is indicative of their evolved diagnostic expertise and mastery of pattern recognition in glaucomatous progression [25].

The reliance on external case references by novices, as noted in some cases, maybe a compensatory mechanism for their relative inexperience. This behavior highlights the gap in their approach to long-term patient management and the development of pattern recognition skills.

### Guidelines for Developing AI System to Minimize Diagnostic Variations

In this section, we propose a set of guidelines for developing an AI system for glaucoma diagnosis based on our findings. These guidelines are intended for a human-AI collaborative environment, where the AI system will be used as a decision-support tool rather than a standalone decision maker. We suggest that by following these guidelines, the AI system will effectively support optometrists with different experience levels, bridging the gap between novices and experts, and ultimately improving patient outcomes through enhanced diagnostic accuracy. The collaborative model will also uphold ethical and professional standards, mitigating any legal, ethical, and professional barriers posed by standalone system. As a result, it will facilitate the effective integration of AI into clinical practice.

### Minimizing the Effect of Limited Data Availability

Our findings show variability in diagnostic concordance between novice and expert optometrists when comprehensive patient histories are not available. These findings suggest that AI systems should have a robust module that provides detailed patient histories. If a patient has limited historical data, the system should present novices with 2 features. It should identify and display data from the most similar patient cases with the same conditions [26] along with the decisions made in that case. It should also highlight the dissimilarities between the selected patient and the current patient. The AI system should be trained with expert decision-making patterns by modeling the diagnostic approaches and visualizing the variables by estimating the missing data [27] backward in time to give them an idea of how history would look. These 2 features would minimize the limited data availability challenge and allow novices to make more accurate and consistent decisions in glaucoma diagnosis.

### Evolving AI System for Initial and Subsequent Visits

Our result shows that in the initial visit, the expert optometrists adopt a comprehensive approach to assess all factors, except they do not carry analytical analysis (any progression and change analysis, and structure and function correlation analysis). As they move to subsequent visits, they still follow a comprehensive approach but with limited focus on patient historical information, instead, their focus shifts to analytical assessment. We accordingly suggest that an AI system should behave in a similar way and evolve along with the visits. In the initial visits, the AI system should prompt a novice to adopt a comprehensive approach and highlight important factors such as family history, and age. In the subsequent visits, the AI system should highlight any analytical changes. It would be better that the AI system present a hierarchy of features, by demonstrating which features require more or less importance to assess during both the initial visit and subsequent follow-up visits. These features of AI system will help novices in understanding the optimal time for any analysis.

### Follow-Up Time Suggestion

The follow-up time analysis shows variation among optometrists. We suggest that for better patient management, the AI system should provide an optimal time frame for follow-up visits [28] which should be based on patient data and clinical indicators. Along with follow-up interval, the system should also display the variables that influence this decision, such as disease progression, patient history, and symptoms. With this feature, we can bring more consistency in follow-up time suggested by optometrists.

### AI System Features and Behavior

Most of the AI systems developed by researchers for glaucoma diagnosis focus on the detection of glaucoma with binary outputs [29-31]. However, to gain optometrists’ trust, the AI system developed for glaucoma should also include a confidence score, include a detailed explanation of how it arrived at that specific decision, and provide a visualization of the risk factors in a hierarchical manner [32,33]. This will ensure that the optometrist does not overlook any important details. We further suggest that in the context of glaucoma, the system should not rely on only one source of data, but that it should combine multiple sources of data as used by optometrists. The optometrist will have more trust if the system uses a similar modal data for decision-making. Additionally, the AI system should have an interactive interface that offers an adjustable variables feature [34] that allows the optometrist to modify the values of any factor and see how different scenarios would affect the outcomes, such as diagnosis, severity, and follow-up time. For example, if the optometrist changes a variable to reflect a worsening condition, the AI system will adjust the follow-up recommendation accordingly. Such an AI system design for glaucoma will ensure more consistent decision-making regardless of expertise level.

### Conclusions

This study investigated the diagnostic decision-making differences between novice and expert optometrists in glaucoma diagnosis, focusing on how these variations influence clinical judgment and patient outcomes. Through in-depth interviews, we identified significant disparities in diagnostic approaches. Experts demonstrated higher concordance rates with limited data, emphasizing their cognitive efficiency and intuitive decision-making, while novices relied more on structured methods and external references. Additionally, there were significant variations between experts and novices in the analysis of different exams and prioritization factors.

Based on our findings we proposed guidelines for a human-AI glaucoma diagnosis system that are aimed to minimize diagnostic variations. Incorporating the recommended features in an AI system, and using a human-AI collaboration model, will not only minimize decision inconsistency but will also improve optometrists’ accuracy and enhance patient care by accelerating the diagnostic process and improving decision-making. In future work, we recommend a phased approach, starting by refining these guidelines to meet local requirements, followed by the development of an AI system based on the refined guidelines, and finally simulating and validating it through real-world data and pilot-testing in clinical environments.

## Data Availability

The clinical data of patients used in the study is obtained from the School of Optometry and Vision Sciences, University of Waterloo. Permission was granted to use the data for this study. We have stored all the anonymized data online in a University of Waterloo Borealis repository [37] with restricted access. For research purposes, the data access can be granted by emailing the corresponding author, after taking the necessary steps required.

## Appendix

Multimedia Appendix 1 Higher resolution version of Figure 1.

Multimedia Appendix 2 Generalized codes that were created during the qualitative data analysis process. Different assessments shown on the right columns represent each code.

Multimedia Appendix 3 Descriptive statistics (mean, SEM, upper CI, lower CI, and SD), including details for novices first visits and subsequent visits, and details for experts first visits and subsequent visits.

## Abbreviations

AI: artificial intelligence
