# Supplementary figures and images for: Diagnostic Decision-Making Variability Between Novice and Expert Optometrists for Glaucoma: Comparative Analysis to Inform AI System Design
Source: JMIR Med Inform. 2025 Jan 29;13:e63109. doi: 10.2196/63109 (PMC11822325; doi:10.2196/63109)

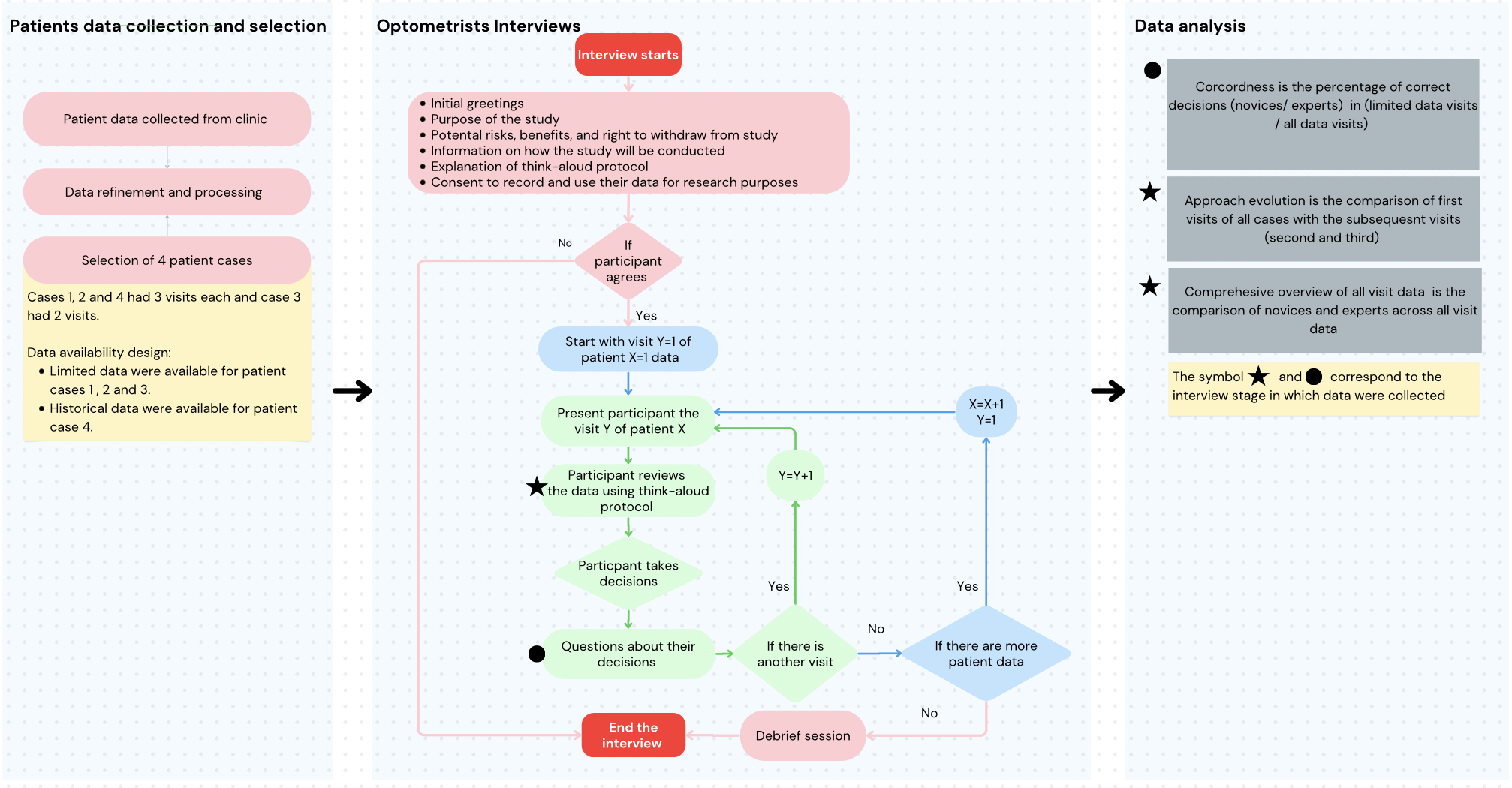

Supplement: Multimedia Appendix 1 [file medinform_v13i1e63109_app1.png]
