# Supplementary material for: Diagnostic Decision-Making Variability Between Novice and Expert Optometrists for Glaucoma: Comparative Analysis to Inform AI System Design
Source: JMIR Med Inform. 2025 Jan 29;13:e63109. doi: 10.2196/63109 (PMC11822325; doi:10.2196/63109)
Supplement: Multimedia Appendix 2 [file medinform_v13i1e63109_app2.docx]

Multimedia Appendix 2

Table S1.

| Family History | Patients having or not having any family history |
| --- | --- |
| Medical History | Blood pressure, diabetes, laser treatments, myopia, sleep apnea, any other treatments |
| Patient Background | Age, ethnicity |
| Clinical Findings | Anterior segment, cataracts, iris, pupil, VA aided/unaided |
| Optic Nerve Exam | Fundus photo analysis, CD ratio, cupping, posterior segment, hemorrhage analysis, ISNT rule, Rim width analysis, OCT findings, RNFL analysis, symmetry findings |
| Optic Nerve Function exam | Visual field findings |
| Structure Function Correlation | OCT and VF correlation, structural and functional vision correlation |
| Progression and Change Analysis | Progression on OCT, Changes in the eyes, color change analysis, defect repeatability analysis, previous visit comparisons |
| Other Risk Factors | Corneal thickness analysis, gonioscopy analysis, Intra ocular pressure |
| Data factors | No data available for comparison, unreliable data, not enough datapoints |
| External factors | Age related consideration, comorbidity consideration, family history consideration, race and ethnicity consideration |
