# Supplementary material for: Diagnostic Decision-Making Variability Between Novice and Expert Optometrists for Glaucoma: Comparative Analysis to Inform AI System Design
Source: JMIR Med Inform. 2025 Jan 29;13:e63109. doi: 10.2196/63109 (PMC11822325; doi:10.2196/63109)
Supplement: Multimedia Appendix 3 [file medinform_v13i1e63109_app3.docx]

Multimedia Appendix 3

Table S1. Novices’ descriptive statistics for first visits and subsequent visits

| **Assessment** | **Visit number** | **Mean** | **Std. Error of Mean** | **95% CI Mean Upper** | **95% CI Mean Lower** | **Std. Deviation** |
| --- | --- | --- | --- | --- | --- | --- |
| Family history | First Visit | 0.788 | 0.072 | 0.93 | 0.646 | 0.415 |
|  | Subsequent Visit | 0.364 | 0.07 | 0.502 | 0.226 | 0.522 |
| Medical history | First Visit | 1.061 | 0.123 | 1.301 | 0.82 | 0.704 |
|  | Subsequent Visit | 0.444 | 0.086 | 0.614 | 0.275 | 0.634 |
| Patient background | First Visit | 0.788 | 0.072 | 0.93 | 0.646 | 0.415 |
|  | Subsequent Visit | 0.296 | 0.063 | 0.419 | 0.173 | 0.461 |
| Clinical findings | First Visit | 0.97 | 0.192 | 1.346 | 0.593 | 1.104 |
|  | Subsequent Visit | 0.509 | 0.088 | 0.682 | 0.337 | 0.639 |
| Optic nerve exam | First Visit | 3.065 | 0.42 | 3.887 | 2.242 | 2.337 |
|  | Subsequent Visit | 1.72 | 0.121 | 1.958 | 1.482 | 0.858 |
| Optic nerve function exam | First Visit | 1.613 | 0.178 | 1.961 | 1.265 | 0.989 |
|  | Subsequent Visit | 1.407 | 0.158 | 1.716 | 1.099 | 1.158 |
| Correlation | First Visit | 0.061 | 0.042 | 0.143 | -0.022 | 0.242 |
|  | Subsequent Visit | 0.127 | 0.058 | 0.242 | 0.013 | 0.433 |
| Progression and Change | First Visit | 0.152 | 0.077 | 0.302 | 8.223×10^-4^ | 0.442 |
|  | Subsequent Visit | 0.352 | 0.084 | 0.517 | 0.187 | 0.619 |
| Other risk factors | First Visit | 2.375 | 0.14 | 2.65 | 2.1 | 0.793 |
|  | Subsequent Visit | 1.512 | 0.1 | 1.707 | 1.317 | 0.637 |

Table S2. Experts’ descriptive statistics for first visits and subsequent visits

| **Assessment** | **Visit number** | **Mean** | **Std. Error of Mean** | **95% CI Mean Upper** | **95% CI Mean Lower** | **Std. Deviation** |
| --- | --- | --- | --- | --- | --- | --- |
| Family history | First Visit | 0.778 | 0.101 | 0.975 | 0.58 | 0.428 |
|  | Subsequent Visit | 0.323 | 0.085 | 0.49 | 0.155 | 0.475 |
| Medical history | First Visit | 1.167 | 0.167 | 1.493 | 0.84 | 0.707 |
|  | Subsequent Visit | 0.452 | 0.121 | 0.689 | 0.214 | 0.675 |
| Patient background | First Visit | 0.944 | 0.056 | 1.053 | 0.836 | 0.236 |
|  | Subsequent Visit | 0.552 | 0.094 | 0.736 | 0.368 | 0.506 |
| Clinical findings | First Visit | 1.118 | 0.169 | 1.449 | 0.786 | 0.697 |
|  | Subsequent Visit | 0.897 | 0.135 | 1.16 | 0.633 | 0.724 |
| Optic nerve exam | First Visit | 2.765 | 0.504 | 3.752 | 1.777 | 2.078 |
|  | Subsequent Visit | 1.926 | 0.261 | 2.438 | 1.414 | 1.357 |
| Optic nerve function exam | First Visit | 1.529 | 0.244 | 2.008 | 1.051 | 1.007 |
|  | Subsequent Visit | 1.179 | 0.212 | 1.595 | 0.762 | 1.124 |
| Correlation | First Visit | 0.111 | 0.076 | 0.261 | -0.038 | 0.323 |
|  | Subsequent Visit | 0.226 | 0.076 | 0.375 | 0.076 | 0.425 |
| Progression and Change | First Visit | 0.167 | 0.121 | 0.404 | -0.071 | 0.514 |
|  | Subsequent Visit | 1.29 | 0.192 | 1.667 | 0.913 | 1.071 |
| Other risk factors | First Visit | 2.222 | 0.191 | 2.596 | 1.849 | 0.808 |
|  | Subsequent Visit | 1.667 | 0.162 | 1.984 | 1.35 | 0.686 |
